# Neuronal Signal Transduction-Involved Genes in Pig Hypothalamus Affect Feed Efficiency as Revealed by Transcriptome Analysis

**DOI:** 10.1155/2018/5862571

**Published:** 2018-12-26

**Authors:** Ye Hou, Mingyang Hu, Huanhuan Zhou, Changchun Li, Xinyun Li, Xiangdong Liu, Yunxia Zhao, Shuhong Zhao

**Affiliations:** ^1^Key Laboratory of Agricultural Animal Genetics, Breeding, and Reproduction of the Ministry of Education and Key Laboratory of Swine Genetics and Breeding of the Ministry of Agriculture, Huazhong Agricultural University, Wuhan 430070, China; ^2^The Cooperative Innovation Center for Sustainable Pig Production, Wuhan 430070, China

## Abstract

Feed efficiency (FE) is an important trait affecting costs in swine industry. Investigation on FE-related genes in different tissues is valuable for molecular breeding. Hypothalamus is a convergent and integrated centre for multiple nutrient-related signals. The present study identified 363 differentially expressed (DE) genes and 14 DE lincRNAs in the hypothalamus of high- and low-FE Yorkshire pigs. Furthermore, 983 significantly correlated DE gene-lincRNA pairs were identified through weighted correlation network analysis (WGCNA) and Pearson correlation analysis. These DE genes were primarily enriched in the neuronal signal transduction process containing the upregulated genes of* VIPR1*,* CCR1*,* CCR5*,* LEPR*,* INSR*,* ADRA1A*,* CCKAR*, and* ADORA3* and the downregulated genes of* GRM1*,* GRM4*,* GRM5*, and* VIPR2*, which were located in the cell membrane. These signal receptors were mainly connected to downstream Jak-STAT signaling that involved the increased genes (*JAK2*,* STAT3*, and* POMC*) and mTOR signaling pathway, including the decreased genes (*CAMKK2*,* AMPK*, and* MTOR*).* STAT3* and* AMPK* genes also played a role in two major hypothalamic neurons of POMC and NPY/AGRP. A total of eight DE lincRNAs also participated in the potential network. In conclusion, neuronal signaling transduction-involved genes and lincRNAs were related to FE variation in pig hypothalamus.

## 1. Introduction

Interests in the pig industry largely depend on feed consumption (>60%). Thus, improving feed efficiency (FE) can effectively reduce costs. Genome-wide association analysis reveals that genetic loci and five candidate genes (*SERPINA3*,* MYC, LEF1*,* PITX2*, and* MAP3K14*) are essential for the feeding behaviour and FE of 338 Duroc boars [1]. Reducing mitochondrial energy metabolism genes (*FABP3*,* RCAN*,* PPARGC1*,* HK2*, and* PRKAG2*) and promoting muscle growth-related genes (*IGF2*,* PDE7A*,* CEBPD*,* PIK3R1*, and* MYH6*) can enhance FE in pigs [2]. Vitamin A metabolism involving key genes (*CYP1A1*,* ALDH1A2*, and* RDH16*) can also affect FE by affecting the energy metabolism in pig liver tissues [3]. The cAMP signaling pathway containing* ATP2B2*,* ATP1A4*, and* VIPR2* is involved in the regulation on FE in pig adipose tissues by affecting lipid metabolism [4].

Hypothalamus is another tissue that can affect FE in cattle and laying duck. FE can be measured by determining the residual feed intake (RFI), which is calculated by the difference between the observed feed intake and the expected feed intake [5]. Animals with a low RFI have a high-FE level [6]. RFI Selection in Angus-sire cattle suggests the differences in hypothalamic neuropeptide gene expression. For instance, the mRNA expression of* NPY* is 64% lower (*p* < 0.05), whereas* POMC* is 350% higher (*p* < 0.01) in the low RFI steers than that of the high-RFI ones [7]. An analysis has also indicated a significant association between the expression levels of hypothalamic neuropeptide genes and FE in laying ducks [8]. These studies have indicated that hypothalamic gene expression plays a potential role in FE variation.

Feed intake is the basic guarantee for the survival of animals and the maintenance of energy balance of the body. Hypothalamic neuropeptides, including NPY, AGRP, and POMC, or peripheral transmitters, such as leptin and insulin, are involved in the regulation of feeding behaviour in mammals [9, 10]. For instance, NPY/AGRP promotes feeding, whereas POMC inhibits feeding behaviour [11]. In addition, leptin can act on the hypothalamus to inhibit the NPY/AGRP anabolic pathway and stimulate the POMC catabolic pathway, leading to reduced feed intake and anorexia [12, 13]. Therefore, hypothalamic neuropeptides or peripheral transmitters regulate the feeding behaviour of mammals.

Long noncoding RNA (lncRNA) can perform diverse biological functions. For example, the lncRNA of Prader–Willi locus can regulate energy balance in mice [14]. Meg3 knockdown modulates insulin synthesis and secretion in mice [15]. Pnky and lncR492 negatively regulate the neural differentiation of murine embryonic stem cells [16, 17]. However, the roles of lncRNAs in the hypothalamus of pig remain largely unknown.

With the increase in the number of genes and lncRNAs, FE has been proposed to be modulated by a complex process. To enhance our understanding on how FE variation is related to hypothalamic gene expression in pigs, we applied high-throughput RNA sequencing (RNA-seq) to identify DE genes and long intergenic noncoding RNAs (lincRNAs) of high- and low-FE pig hypothalamus. Gene ontology (GO) and pathway analysis revealed that neuronal signaling transduction genes in the hypothalamus underlie FE variation by regulating the feed intake of pigs.

## 2. Materials and Methods

### 2.1. Animals and Tissues

The feed intake of 236 purebred castrated Yorkshire boars was detected by applying an ACEMA 64 automated individual feeding system at the Agricultural Ministry Breeding Swine Quality Supervision Inspecting and Testing Centre (Wuhan, China) [2, 3]. FE was analysed for each individual in accordance with previously described methods [2], and three high-FE pigs and three low-FE pigs that significantly differed were selected (*p *< 0.05). Six 90 kg pigs were slaughtered in accordance with the guidelines of the Regulation of the Standing Committee of Hubei People's Congress (Hubei Province, China), and hypothalamic tissues were sampled and snap frozen in liquid nitrogen. All of the experimental protocols were approved by the Ethics Committee of Huazhong Agricultural University (HZAUMU2013-0005).

### 2.2. RNA Library Preparation and Data Analysis

Total RNA was extracted from frozen hypothalamic tissues by using TRIzol reagent (Invitrogen, USA) and sent to Genergy Biotechnology (Shanghai, China) for library construction. However, one of the high-FE samples failed to construct a library, and the five remaining samples (two high-FE samples and three low-FE samples) were applied for follow-up sequencing. After quality was controlled, Illumina HiSeq was performed for RNA-seq. These samples with original transcriptome pair-end data were controlled using FastQC (FastQC: a quality control tool for high-throughput sequence data, http://www.bioinformatics.babraham.ac.uk/projects/fastqc/). TopHat (version 2.1.1) [18] was utilized to align reads to pig reference genome* Sus scrofa *10.2 and* S. scrofa* 11.1, which were downloaded from Ensembl (http://www.ensembl.org/info/data/ftp/index.html). For lincRNA transcript identification, we compared the mapped reads in the intergenic region with the annotated lncRNAs [19].

### 2.3. Differential Expression and Correlation Analysis

The read count of the annotated genes and lincRNAs located in the exon regions was calculated with Htseq-count software [20]. DEseq2 was performed to normalize the expression profile of all of the expressed genes and lincRNAs and to identify the DE gene and lincRNA [21]. The threshold for the selection of DE gene and lincRNA was set as |log_2_FC| > 1 and false discovery rate (FDR) < 0.05 between high- and low-FE pigs. WGCNA was performed to identify the correlated DE gene-lincRNA pairs in the R environment [22]. The R package WGCNA was applied to construct the weight coexpression network on the account of the normalized count matrix from DEseq2 with the soft threshold of 7. The DE gene-lincRNA pairs were also determined by Pearson correlation analysis with the criteria of* p *< 0.05.

### 2.4. Quantitative Real-Time PCR (qPCR) Analysis

The total RNA of the hypothalamus was reverse transcribed into cDNA by utilizing the RevertAid First Strand cDNA synthesis kit (K1621, Thermo Scientific, USA), and the oligonucleotide primers of DE genes and lincRNAs were designed with Oligo7. The primer sequences of DE genes and lincRNAs are listed in Table S1. The relative expression levels of DE genes and lincRNAs in the hypothalamus were quantified through qPCR with* YWHAZ* as a separate internal control [23]. qPCR assay was conducted on a Bio-Rad CFX384 Real-Time System with a SYBR Green PCR Master Mix reagent (Toyobo, Japan) in accordance with the instruction's manual. Reactions were done thrice in 384-well plates, and each well contained 5 *μ*L of 2× SYBR Green PCR Master Mixture, 0.2 *μ*L of forward and reverse primers, 1 *μ*L of template cDNA, and 3.6 *μ*L of RNase-free water. The samples were preincubated at 95°C for 5 min and subjected to 40 PCR amplification cycles (95°C for 30 s, 60°C for 30 s, and 72°C for 20 s). The relative gene and lincRNA expression levels of qPCR data were analysed using the 2^-ΔΔCT^ method, and the statistical analysis method used for DE analysis was presented in Table S2. Significance level was set at* p* < 0.05.

### 2.5. GO Enrichment and Pathway Analysis

The human homologous Ensembl Gene IDs of the identified DE genes were applied because their functional annotation information was more complete than that of the pig. Gene enrichment in GO biological processes and pathways was performed with the DAVID Bioinformatics Resources version 6.8 (http://david.abcc.ncifcrf.gov/) with a cut-off criterion of* p *< 0.01 [24]. The potential network was visualized with Cytoscape [25].

## 3. Results

### 3.1. RNA-Seq Data Mapping and Annotation

**Figure 1:**
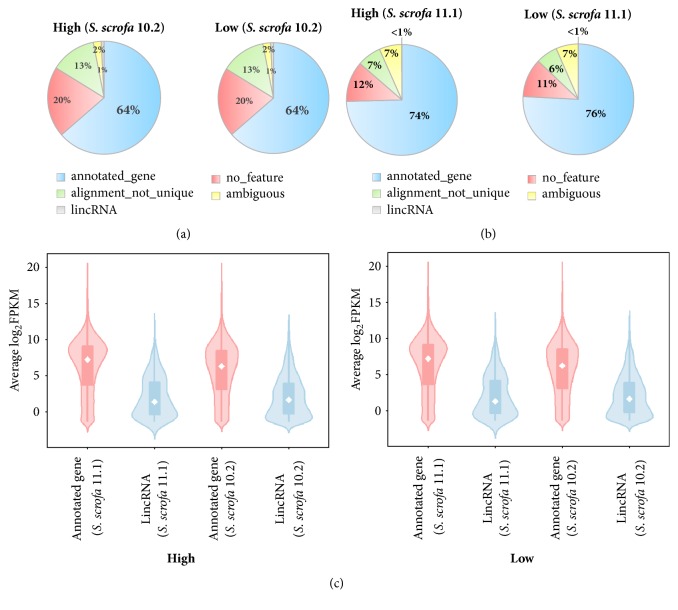
Annotation of the mapped reads of RNA-seq data in the hypothalamic tissue on two porcine genome versions. Distribution of the average mapped reads of high- and low-FE pigs on porcine* S. scrofa* 10.2 genome (a) and* S. scrofa* 11.1 genome (b). Expression patterns of annotated genes and lincRNAs in the high- and low-FE groups on the two porcine genome versions (c).

The RNA of the hypothalamic tissues was extracted for RNA-seq, and their clean data were deposited to the NCBI Sequence Read Archive (SRA) under the series SRP149276. After the adaptors were removed and filtered, approximately 80% of the clean reads were mapped to the porcine genome, and uniquely mapped reads reached 90% on the* S. scrofa* 10.2 genome, whereas the mapped rate on the* S. scrofa* 11.1 genome was higher than that of the other genome (Table S3). The mapped reads were further annotated on two porcine genome versions. Among them, over 60% was located in the annotated gene region, and approximately 1% was aligned to lincRNA on the porcine* S. scrofa* 10.2 genome (Figure 1(a)). In comparison with the old genome version (*S. scrofa* 10.2), upstream of 70% of the reads was aligned to the annotated genes and less than 1% to lincRNA on the porcine* S. scrofa *11.1 genome (Figure 1(b)). The expression patterns of the annotated genes and lincRNAs were scaled by log_2_FPKM (fragments per kilo base of exon per million fragments mapped [FPKM]) in the high-and low-FE groups on the two porcine genome versions, indicating that the average expression of the annotated genes was higher than those in lincRNAs in the two groups and the two porcine genome versions (Figure 1(c)).

### 3.2. Differences in the Expression of Annotated Genes and lincRNAs in High- and Low-FE Pigs

**Figure 2:**
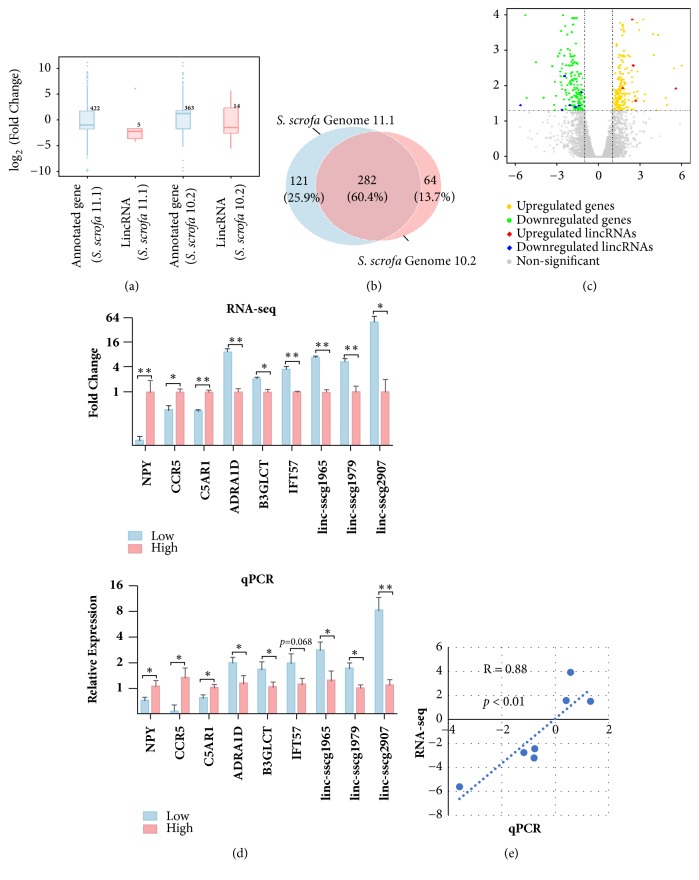
Differential expression analysis in the high- and low-FE pigs. (a) Differential expression patterns of annotated genes and lincRNAs on the two porcine genome versions scaled by log_2_FC (|log_2_FC| > 1, FDR < 0.05). (b) Comparison of the Venn diagram of DE genes between two genome versions. (c) Plot of the DE-annotated genes and lincRNAs with the threshold of |log_2_FC| > 1, FDR < 0.05. The x- and y-axes represent log_2_FC and log_10_(*p *value), respectively. (d) The RNA-seq and qPCR verification of DE genes and lincRNAs in the hypothalamic tissue of high- and low-FE pigs (n = 12, 6 high-FE pigs versus 6 low-FE pigs); *∗* represents* p *< 0.05 and *∗∗ p *< 0.01. (e) Correlation analysis of RNA-seq data and qPCR results with log_2_FC value. log_2_FC, log_2_Fold Change (FE high/low).

In this RNA-seq study, the differential expression patterns of the annotated genes and lincRNAs were analysed. The DE genes on the two porcine genome versions had a similar amount (422 for* S. scrofa* 11.1 and 363 for* S. scrofa* 10.2), whereas more DE lincRNAs were derived from the* S. scrofa* 10.2 genome version (5 for* S. scrofa* 11.1, and 14 for* S. scrofa* 10.2).* S. scrofa* 10.2 genome version was more evenly distributed in the high- and low-FE groups than its counterpart (|log_2_FC| > 1, FDR < 0.05, Figure 2(a)), because the* S. scrofa* 10.2 genome version has a more comprehensive lincRNA annotation [19]. The comparison with DE genes on the two genome versions demonstrated that the DE genes showed a similar trend, and over 60% of the DE genes overlapped on the two genome versions (Figure 2(b)). Thus, a follow-up analysis was based on the* S. scrofa* 10.2 genome version in which 377 significantly DE transcripts, including 363 annotated genes, and 14 lincRNAs, were identified in the hypothalamus. Among them, 188 DE genes and 5 DE lincRNAs were upregulated, whereas 174 DE genes and 9 DE lincRNAs were downregulated in the high-FE pigs compared with those in the low-FE pigs (Figure 2(c)). Moreover, top 10 DE genes and lincRNAs are provided in Table 1.

qPCR was further applied to validate DE genes and lincRNAs in two-group samples, which were identified by RNA-seq data, and performed on six high-FE pigs and six low-FE pigs with significantly different phenotypes [2], including individuals for RNA-seq. Moreover, six DE genes (*NPY*,* CCR5*,* C5AR1*,* ADRA1D*,* B3GLCT*, and* IFT57*) and three DE lincRNAs (linc-sscg1965, linc-sscg1979, and linc-sscg2907) were randomly selected to validate the sequencing data by* YWHAZ* normalization. The qPCR results were similar to RNA-seq (Figure 2(d)). Furthermore, the correlation coefficient (R) was 0.88, with highly significant consistency between two methods (Figure 2(e)).

### 3.3. Identification of the Correlated Expression of DE Gene-lincRNA Pairs

**Figure 3:**
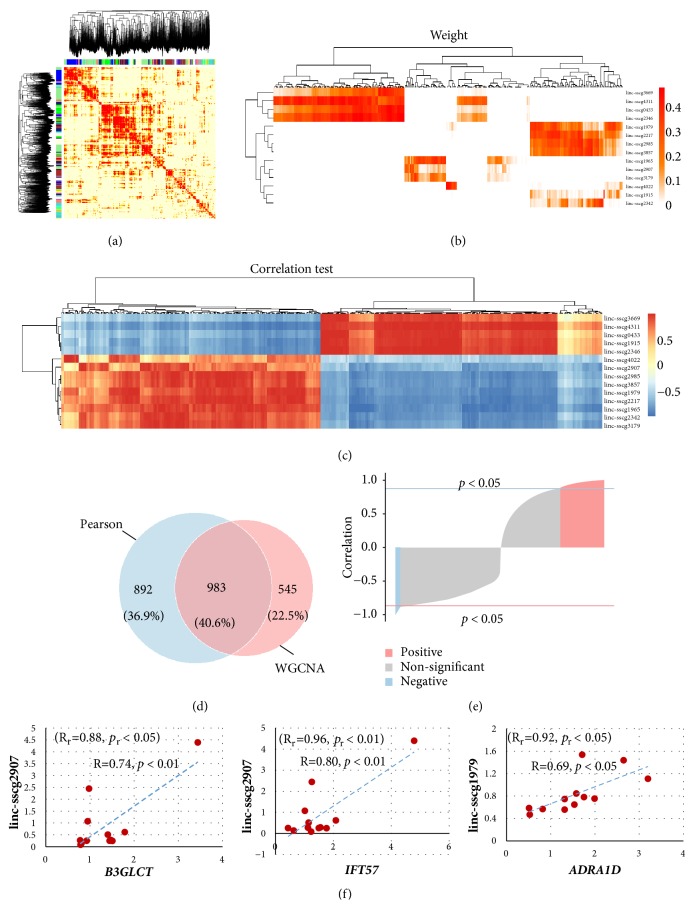
Correlation analysis of DE genes and lincRNAs in the hypothalamus of high- and low-FE groups. (a) WGCNA modules of DE gene-lincRNA expression patterns. (b) Correlation analysis between DE genes (abscissa axis) and lincRNAs (vertical axis) with WGCNA. In the colour bar, the darker the colour is, the more relevant the gene is. (c) Heat map of correlation analysis with the Pearson correlation coefficient between DE genes and lincRNAs. Positive and negative correlations are represented by red and blue, respectively. (d) Venn diagram of the WGCNA and Pearson correlation of DE gene-lincRNA pairs. (e) Pearson correlation coefficient analysis of DE gene-lincRNA pairs. Positive and negative correlated pairs are represented by red and blue, respectively. (f) The correlation of DE gene-lincRNA pairs was identified by qPCR. The value of R_r_ and *p*_r_ represents RNA-seq results.

To understand the potential function of lincRNA, we analysed the correlation of DE genes and lincRNAs through WGCNA and Pearson correlation analysis. All of the genes and lincRNAs were divided into 19 modules by WGCNA (Figure 3(a)). Among them, seven modules containing DE lincRNAs were extracted to detect DE gene-lincRNA weights in the same modules, which totally identified 1528 DE gene-lincRNA pairs, including 328 DE genes and 14 DE lincRNAs (Figure 3(b)). Furthermore, 1875 DE gene-lincRNA pairs were filtered on the basis of the analysis of the Pearson correlation coefficient with a threshold of* p* < 0.05 (Figure 3(c)). A total of 983 correlated DE gene-lincRNA pairs (over 40%) coexisted in the two methods from Venn diagram (Figure 3(d)). Among them, 886 (90.13%) were positively correlated (*p *< 0.05; Figure 3(e)). Besides, three DE gene-lincRNA pairs were randomly selected to identify the correlation by qPCR methods (n=12). The results showed that the R value of DE gene and relative lincRNA was around 0.7 with a high correlation (*p* <0.05), which was consistent with the sequencing data (Figure 3(f)).

### 3.4. Function Annotation

**Figure 4:**
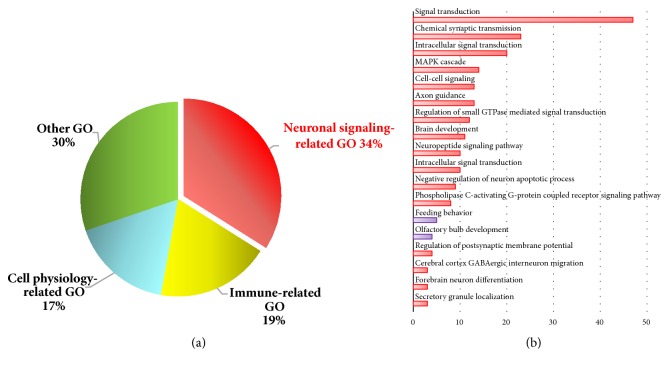
Significantly enriched GO terms for the biological process of DE genes in the hypothalamus of high- and low-FE pigs. (a) Classification of significantly enriched GO terms. Red, yellow, blue, and green represent neuronal signaling process, immune-related terms, cell physiology-related terms, and other GO terms, respectively. (b) Eighteen detailed GO terms from neuronal signaling-related GO.

The DE genes were subjected to function enrichment analysis to determine significant biological processes with DAVID Bioinformatics Resources (version 6.8). A total of 190 GO terms were available (EASE Score, 0.1). Among them, 53 were significantly enriched (*p* < 0.01, Figure S1). GO terms based on similar biological functions were clustered into four major categories: neuronal signaling-related, immune-related, cell physiology-related, and other GO terms. Among these categories, neuronal signaling-related GO terms possessed the largest proportion (18, 34%; Figure 4(a)), and 18 detailed GO terms are listed in Figure 4(b). Signal transduction was the most enriched pathway containing 47 DE genes. Feed intake-related GO terms comprising feeding behaviour pathway and olfactory bulb development pathway were also enriched (Figure 4(b)).

### 3.5. Potential Network of DE Genes and LincRNAs Related to FE in Pig Hypothalamic Tissue

**Figure 5:**
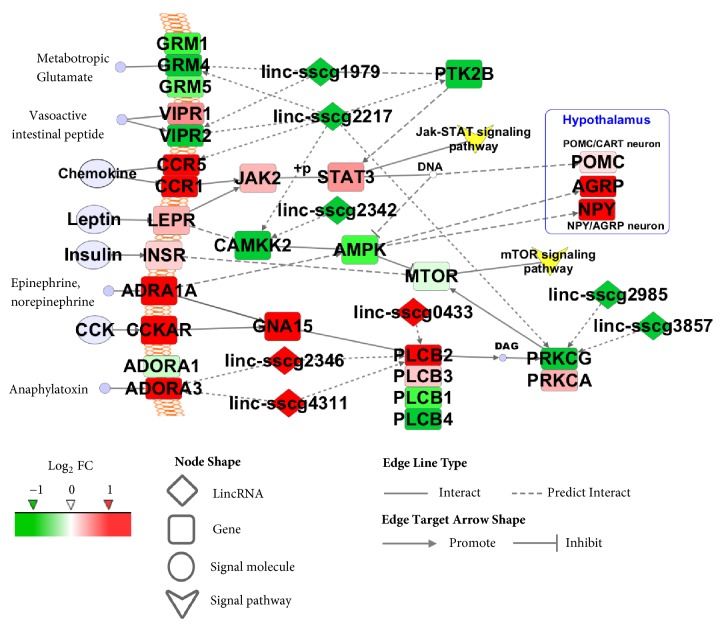
Key network of DE genes and lincRNAs in the hypothalamus of high- and low-FE pigs analysed with Cytoscape. Red and green correspond to upregulation and downregulation in high-FE pigs.

The key network related to FE in the pig hypothalamus was investigated using Cytoscape to integrate the potential interaction between DE genes and lincRNAs (Figure 5). Functional enrichment analysis revealed that signal transduction was the most important pathway that corresponded to the most enriched neuroactive ligand-receptor interaction pathway in the KEGG analysis involving the upregulated genes, namely,* VIPR1*,* CCR1*,* CCR5*,* LEPR*,* INSR*,* ADRA1A*,* CCKAR*, and* ADORA3*, and downregulated genes, such as* GRM1*,* GRM4*,* GRM5*, and* VIPR2*, which were located in the cell membrane. Most of them were upregulated in the high-FE pigs. Moreover, these signal transduction genes mainly communicated with downstream Jak-STAT signaling involving the upregulated genes, namely,* JAK2*,* STAT3*, and* POMC*. These signal transduction genes also interacted with the mTOR signaling pathway, including the downregulated genes, such as* CAMKK2*,* AMPK*, and* MTOR*.* STAT3* and* AMPK* genes also affect POMC and NPY/AGRP, which are the two major hypothalamic neurons. Furthermore, eight DE lincRNAs were implicated in the potential network with a correlation threshold of |R| > 0.95 and* p* < 0.01. Among these lincRNAs, linc-sscg2217 possessed the most potential target genes (*PTK2B*,* CCR5*,* GRM4*,* VIPR2*,* CAMKK2*, and* PRKCG*), which were downregulated in the high-FE pigs. Therefore, neuronal signal transduction-involved genes and lincRNAs were associated with FE in the pig hypothalamus.

## 4. Discussion

The profitability of the pig industry largely depends on FE improvement. Thus, genes related to FE should be identified for molecular breeding. Feeding behaviour is mainly controlled by the hypothalamus, as a vital brain region [26, 27]. The present study identified 363 DE genes and 14 DE lincRNAs in the high- and low-FE pig hypothalamus. The DE genes were subjected to functional enrichment analysis and clustered into four major categories based on similar biological functions: neuronal signaling-related, immune-related, cell physiology-related terms, and other GO terms. Among these categories, neuronal signaling-related GO terms possessed the largest proportion (18, 34%), and feed intake-related GO terms involving feeding behaviour pathway and olfactory bulb development pathway were also enriched. Therefore, neuronal signaling-related GO terms might affect FE.

The hypothalamic neural system participates in feed intake regulation, including modulating feed-related signals, such as cholecystokinin (CCK), anaphylatoxin, glutamate, leptin, insulin, chemokine, vasoactive intestinal polypeptide, and anaphylatoxin. CCK release promotes feed termination by activating vagal afferent neurons [28]. Our study showed that* CCK* and its receptor* CCKAR* were upregulated in the high-FE pigs.* LEPR* and* INSR*, expressed by brain neurons, can reduce feed intake [12]. In our study,* LEPR* and* INSR* were upregulated in the high-FE pigs. The metabotropic glutamate receptors* GRM1* and* GRM5*, which can stimulate feeding in rats and mice [29, 30], were downregulated in our high-FE pigs. Inflammatory factors, such as chemokine, function as a negative regulator of feed intake and weight maintenance [31, 32]. Chemokine receptors, including* CCR1* and* CCR5*, were upregulated in our high-FE pigs. In general, the amount of these signal receptors that promote appetite decreases, whereas the amount of those that inhibit feed intake increases in the high-FE group. However, to maintain the energy balance of the body, several genes showed an opposite expression trend. For instance, VIP inhibits feeding in mice [33] and chicks [34] and is widely expressed in the nervous systems of vertebrates. Our results indicated that VIP played a bidirectional role involving two receptors, namely, the increased* VIPR1* and the decreased* VIPR2*, in the high-FE pigs. Therefore, hypothalamic neural signaling involved genes related to feed intake and their different expression levels of the high- and low-FE pigs might further affect FE.

Downstream genes and their relative pathways interacting with neural signals were also related to feed intake in the high- and low-FE pigs. The appetite regulatory hormone leptin and the inflammatory factor chemokine are involved in the JAK-STAT pathway and regulate feed intake [35]. The current study indicated that the upregulated genes of* LEPR*,* CCR1*,* JAK2*, and* STAT3* acted on the POMC neuron, whereas the genes of* CAMKK2*,* AMPK*, and* MTOR* in the mTOR signaling pathway were downregulated in the high-FE pigs. Leptin can modulate hypothalamic mTOR signaling; with this ability, leptin can inhibit rat feed intake [36]. This finding is also consistent with the present results. The upregulation of* AMPK* in the hypothalamus increases feed intake and body weight [37], and this phenomenon is in line with the downregulation of* AMPK* in the high-FE pigs to inhibit feeding. Furthermore, improving AGRP neuron electrical activity can elicit feeding, whereas stimulating the POMC neuron negatively regulates feeding [11]. The current study indicated that* AGRP* and* POMC* were upregulated in the high-FE pigs and likely implicated in keeping the body energy balance. Besides, our previous studies demonstrated that mitochondrial energy metabolism in the muscle, vitamin A metabolism in the liver, and lipid metabolism in adipose tissues are related to energy metabolism, which also have effect on FE in pigs [2–4]. Therefore, hypothalamic tissues affected feed intake through neuronal signal transduction-involved genes, which were related to the energy metabolism of other tissues, such as muscle, liver, and adipose tissues, collectively leading to variations in FE in pigs.

In addition, 14 DE lincRNAs were found in the high- and low-FE pigs. Linc-sscg2217 and linc-sscg2342 were downregulated in the hypothalamus of high-FE pigs and were positively correlated with* CAMKK2*, which participated in the AMPK pathway.* CAMKK2* in the hypothalamus regulates feed intake, and a decrease in its expression can suppress the feed intake of mice and eventually reduce their body weight [38]. The downregulated DE gene* PRKCG* was targeted by three decreased DE lincRNAs, namely, linc-sscg2217, linc-sscg2985, and linc-sscg3857, in the high-FE groups, involving the insulin-mediated inhibition of neuronal necrosis [39]. Other DE lincRNAs (linc-sscg1979, linc-sscg2217, linc-sscg2346, and linc-sscg4311) related to signal receptors (*GRM4*,* VIPR2*,* CCR5*,* ADRA1A*,* ADORA3*, and* C5AR1*) might also participate in modulating neuron signal transduction. These studies suggested that these genes related to neuronal signal transduction affect feed intake. This current research indicated that neuronal signal transduction-involved genes potentially influence FE by modulating feed intake. Collectively, neuronal signaling transduction-involved genes in the hypothalamus could affect FE variations by regulating the feed intake of pigs.

## Figures and Tables

**Table 1:** Top 10 DE genes and lincRNAs in the high- and low-FE pigs.

**Source**	**ID**	**Gene Name**	**log**_**2**_ **FC(FE_H/L)**	***p*-Value**	**FDR**
**Annotated gene**	ENSSSCG00000007164	*OXT*	11.05	7.98E-18	2.61E-14
	ENSSSCG00000007163	*AVP*	10.41	1.08E-13	1.46E-10
	ENSSSCG00000023462	*GPR50*	9.11	4.18E-13	5.25E-10
	ENSSSCG00000017410	*HCRT*	8.92	1.82E-38	2.97E-34
	ENSSSCG00000000858	*PMCH*	8.70	1.15E-09	8.92E-07
	ENSSSCG00000003430	*NPPA*	-9.88	3.11E-06	7.36E-04
	ENSSSCG00000014415	*GPR151*	-9.24	6.03E-05	6.56E-03
	ENSSSCG00000028079	*RPL15*	-7.86	7.64E-07	2.31E-04
	ENSSSCG00000013724	*AC018761.5*	-5.29	2.34E-07	1.01E-04
	ENSSSCG00000003170	*SLC17A7*	-5.19	3.51E-04	2.23E-02

**LincRNA**	linc-sscg3669	linc-sscg3669	5.58	1.34E-04	1.20E-02
	linc-sscg0433	linc-sscg0433	2.68	4.45E-04	2.65E-02
	linc-sscg2346	linc-sscg2346	2.50	1.76E-05	2.67E-03
	linc-sscg4311	linc-sscg4311	2.43	3.80E-07	1.35E-04
	linc-sscg1915	linc-sscg1915	1.74	1.28E-04	1.15E-02
	linc-sscg2907	linc-sscg2907	-5.64	6.71E-04	3.53E-02
	linc-sscg4022	linc-sscg4022	-5.06	1.67E-07	7.47E-05
	linc-sscg1965	linc-sscg1965	-2.78	3.03E-14	4.50E-11
	linc-sscg3179	linc-sscg3179	-2.64	1.09E-03	4.83E-02
	linc-sscg1979	linc-sscg1979	-2.45	4.36E-05	5.28E-03

## Data Availability

The RNA-seq data of the hypothalamic tissues used to support the findings of this study have been deposited in the NCBI Sequence Read Archive repository (SRP149276).

## Supplementary Materials

Supplementary Materials Figure S1: cartogram of DE gene-enriched GO terms (*p* < 0.01). Table S1: the qPCR primer sequences of DE genes and DE lincRNAs. Table S2: the DE analysis of qPCR results. Table S3: summary of RNA-seq data from five hypothalamus samples.
