# Supplementary material for: Neuronal Signal Transduction-Involved Genes in Pig Hypothalamus Affect Feed Efficiency as Revealed by Transcriptome Analysis
Source: Biomed Res Int. 2018 Dec 26;2018:5862571. doi: 10.1155/2018/5862571 (PMC6327278; doi:10.1155/2018/5862571)
Supplement: Supplementary Materials — Figure S1: cartogram of DE gene-enriched GO terms (p < 0.01). Table S1: the qPCR primer sequences of DE genes and DE lincRNAs. Table S2: the DE analysis of qPCR results. Table S3: summary of RNA-seq data from five hypothalamus samples. [file 5862571.f1.docx]

**Neuronal signal transduction-involved genes in pig hypothalamus affect feed efficiency as revealed by transcriptome analysis**

**Neuronal signal transduction in pig hypothalamus**

Ye Hou^1^, Mingyang Hu^1^, Huanhuan Zhou^1^, Changchun Li^1,2^, Xiangdong Liu^1,2*^ , Yunxia Zhao^1*^, Xinyun Li ^1,2^ , Shuhong Zhao^1,2^

^1^ Key Laboratory of Agricultural Animal Genetics, Breeding, and Reproduction of the Ministry of Education and Key Laboratory of Swine Genetics and Breeding of the Ministry of Agriculture, Huazhong Agricultural University, Wuhan 430070, P. R. China.

^2^ The Cooperative Innovation Center for Sustainable Pig Production, Wuhan 430070, P. R. China

Correspondence should be addressed to Xiangdong Liu, liuxiangdong@mail.hzau.edu.cn; and Yunxia Zhao, yxzhao@mail.hzau.edu.cn


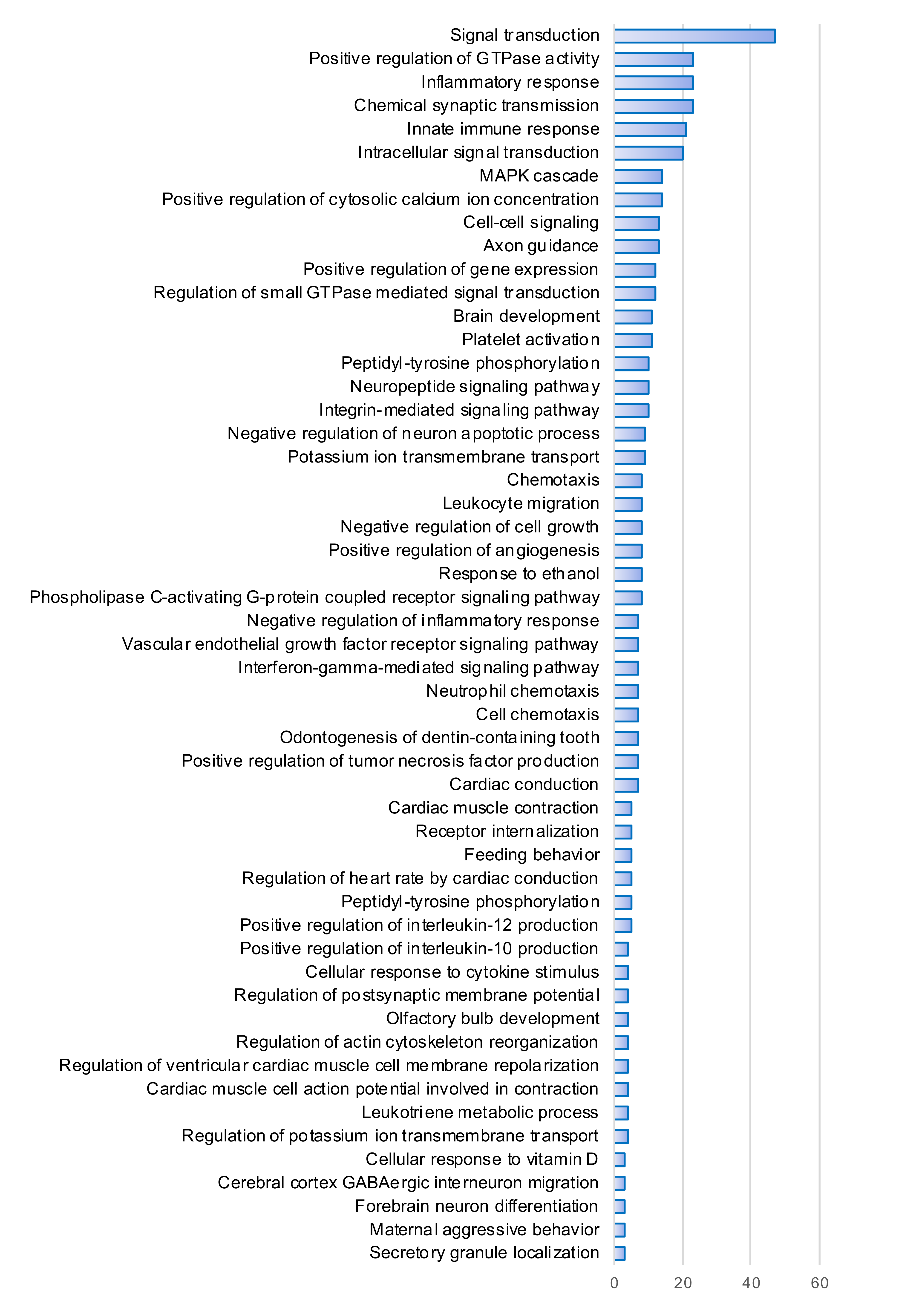


**Figure S1** Cartogram of DE gene-enriched GO terms (*p* < 0.01).

**Table S1** qPCR primer sequences of DE genes and DE lincRNAs.

| Gene | Forward | Reverse |
| --- | --- | --- |
| *NPY* | AAACGATCTAGCCCCGAGAC | TCACTTCCCATCACCACACAG |
| *CCR5* | GAGGAGCTGAGACATCCGTTC | AAACCCGAAGATGAACACCAGT |
| *C5AR1* | AGAACCTCGGAGGGACCAGA | ACTCCCACCAGGAAGACAATG |
| *ADRA1D* | CCGAAGCTCACTGTCCCTAC | GGGTAGATGAGCGGGTTCAC |
| linc-sscg1965 | CTCCCCAGAATGCGTTGTCA | ACAGCTGGTCTTTCCCTCTCT |
| linc-sscg1979 | CGAAAGTTCCTCGGATCACC | ATCTTACCCGTTCCCATACCAG |
| linc-sscg2907 | GGCCGTCGCATATTCAGTCT | CCCTGCGAAATAGCTTCCCT |
| *YWHAZ* | TGATGATAAGAAAGGGATTGTGG | GTTCAGCAATGGCTTCATCA |

**Table S2** The DE analysis of qPCR results.

| Name | Standard normal distribution | Test for homogeneity of variances | Test | *p* value |
| --- | --- | --- | --- | --- |
| *NPY* | Yes | No, Homogeneity of variances transformation | Student’s *t* test | 0.048 |
| *CCR5* | Yes | No, Homogeneity of variances transformation | Student’s *t* test | 0.046 |
| *C5AR1* | Yes | Yes | Student’s *t* test | 0.023 |
| *ADRA1D* | Yes | Yes | Student’s *t* test | 0.030 |
| *B3GLCT* | No | No | Wilcox test | 0.047 |
| *IFT57* | Yes | Yes | Student’s *t* test | 0.068 |
| linc-sscg1965 | No | No | Wilcox test | 0.041 |
| linc-sscg1979 | Yes | Yes | Student’s *t* test | 0.011 |
| linc-sscg2907 | No | No | Wilcox test | 0.0021 |

**Table S3** Summary of RNA-seq data from five hypothalamus samples

| Group | Sample | Input | Total mapped | | Uniquely mapped | |
| --- | --- | --- | --- | --- | --- | --- |
|  |  |  | (*S. scrofa* 10.2) | (*S. scrofa* 11.1) | (*S. scrofa* 10.2) | (*S. scrofa*11.1) |
| High FE | H1 | 17502515 | 14168828  (80.95%) | 15897597  (90.83%) | 13211884  (93.25%) | 15634042  (98.34%) |
|  | H2 | 17025069 | 14267762  (83.80%) | 15837861  (93.03%) | 13369448  (93.70%) | 15528300  (98.05%) |
| Low FE | L1 | 17308544 | 14511380  (83.84%) | 16156762  (93.35%) | 13572456  (93.53%) | 15907003  (98.45%) |
|  | L2 | 17050747 | 13869388  (81.34%) | 15729587  (92.25%) | 12867723  (92.78%) | 15517877  (98.65%) |
|  | L3 | 18882277 | 15482469  (81.99%) | 17328399  (91.77%) | 14435533  (93.24%) | 17008462  (98.15%) |
